# Homocysteine, Carotid Intima Media Thickness and NIHSS Score: Clinical Relevance in Indian Stroke Patients

**DOI:** 10.22088/cjim.15.2.259

**Published:** 2024

**Authors:** Vatsal Navin Jain, Priyanka Rana, Kshitij Arun Bhoge, Swati Ghanghurde, Mahesh B Phad, Mohit Vijay Rojekar

**Affiliations:** 1 Rajiv Gandhi Medical College & Chhatrapati Shivaji Maharaj Hospital, Kalwa, Thane, India; 2 Department of Biochemistry, Rajiv Gandhi Medical College & Chhatrapati Shivaji Maharaj Hospital, Kalwa, Thane, India; 3 Department of Pathology,Rajiv Gandhi Medical College & Chhatrapati Shivaji Maharaj Hospital, Kalwa, Thane, India; 4 Department of Biochemistry, Government Medical College, Baramati, India

**Keywords:** Homocysteine, Carotid intima-media thickness, Stroke

## Abstract

**Background:**

Stroke is one of the leading causes of mortality and morbidity worldwide accounting for 85% of global deaths from stroke. This study aimed to evaluate the role of homocysteine (HCY) in modulating various stroke parameters and it’s with carotid intima-media thickness (IMT).

**Methods:**

78 patients of radiology-confirmed acute ischemic stroke were recruited for this study and National Institutes of Health Stroke Scale (NIHSS) score was evaluated upon admission. Blood samples were tested for serum HCY, fasting blood glucose (FBG) and lipid profile. Ultrasonography of neck ascertained IMT of Common (CCA) and internal carotid artery (ICA).

**Results:**

Average age of male and female subjects was 57.88 ± 13.97 & 59.16 ± 13.62 years respectively. 71.93% of stroke patients were hyperhomocysteinemic (HHcyc) and 24.36% were hyperlipidemic. Patients with NIHSS ≥ 5 had higher low-density lipoprotein cholesterol (LDLC) than those with NIHSS < 5. HCY cutoff of ≥ 15 μmol/L had 91.7% sensitivity & 66.7% specificity for predicting. HHcyc state was associated with increased ICA IMT. HHcyc state was best predicted by ICA IMT with which it is positively correlated (P-Value = 0.012).

**Conclusion:**

HHcyc state holds a good predictive value for severity of stroke. We also came to a conclusion that ICA IMT measurement may also reduce the need for a homocysteine test as it predicts higher HCY levels; this will reduce the burden on resources. We suggest that evaluating HCY and ICA IMT should be made part of the standard protocol for management of stroke.

A cerebrovascular accident (CVA), or stroke, as it is popularly known, is one of the leading causes of mortality and morbidity worldwide (1) with developing countries accounting for 85% of global deaths from stroke (2). CVA is a medical emergency presenting with an abrupt onset of neurological deficit that can be attributed to a focal vascular cause. Thus, the definition of stroke is clinical, and laboratory studies including brain imaging are used to support the diagnosis.

The clinical manifestations of stroke are highly variable because of the complex anatomy of the brain and its vasculature. Establishing the severity of stroke in a patient helps to gauge prognosis and also shapes therapeutic approaches. Hence, various scales have been developed to evaluate the clinical severity of stroke. Among these, the National Institutes of Health Stroke Scale (NIHSS) is the most comprehensive and is easy to perform at bedside. (3) Muir et al.’s study (4) among 373 patients of acute ischemic stroke showed that at a 3-month follow-up period, the median baseline NIHSS score of patients who were alive at home (good prognosis) was 4, patients alive in care (moderate prognosis) was 14, and dead patients (poor prognosis) was 18. This forms the basis of the grading system adopted by us. 

Measurement of the carotid intima-media thickness (IMT) is an upcoming tool in research methodology. IMT measurement in stroke patients is particularly useful as it gives radiological evidence for atherosclerosis and helps to gauge both - treatment aggressiveness, i.e. dosage of hypolipidemic drugs, anticoagulants etc., as well as prognosis, such as risk of recurrence - when considered along with traditional risk factors. Since the late 1990s, many factors influencing IMT have been identified. Darabian et al. (5) reviewed the significance of carotid IMT in clinical research and found that IMT was affected by most cardiovascular risk factors like age, total Cholesterol, high-density-lipoprotein cholesterol, smoking, Diabetes Mellitus (DM), Hypertension (HTN) etc. In addition to traditional cardiovascular risk factors, there are novel factors under consideration such as homocysteine.

 Plasma homocysteine (HCY) levels are affected by both acquired and genetic factors. Acquired factors include ageing, smoking, impaired renal function, and medication with drugs such as corticosteroids and cyclosporine, and the main genetic ones are classical homocystinuria and C677T homozygote mutation of the 5,10-methylenetetrahydrofolate reductase (*MTHFR*) gene (6, 7).

Normal fasting serum/plasma homocysteine levels remain below 15 μmol/L. We have referred to this state as Euhomocysteinemia (EHcy). Hyperhomocysteinemia (HHcy) is the elevation of fasting homocysteine levels beyond 15 μmol/L. High plasma levels of HCY have been implicated in the development of vascular diseases, including stroke. Elevated serum HCY is described to have an atherosclerotic and thrombotic effect by various mechanisms such as homocysteinylation, induction of oxidative stress and excitotoxicity (8, 9). Over the last decade, convincing evidence has been gathered on the relation between elevation of plasma HCY and ischemic stroke. Though HCY has been studied extensively, the evidence supporting its use in practice is conflicted vis-a-vis its prognostic value.

The World Health Organization estimates that by 2030, 80% of all strokes will occur in low and middle-income countries (10). Hence, it is imperative to investigate novel risk factors for stroke and their management thoroughly. Our study aimed to evaluate the role of HCY in modulating various stroke parameters. The primary objective was to study the correlation of HCY levels with carotid IMT in stroke patients and investigate if HCY levels had any predictive value for the NIHSS score.

## Methods

**Study design:** This study adheres to the observational cohort guidelines as specified in the STROBE checklist (11). A cross-sectional, observational study was conducted in a tertiary healthcare center in the state of Maharashtra in India from July to August 2022 after obtaining approval from the Institutional Clinical Ethics Committee (letter no. IEC/A/264/06/2022 dated 27/06/2022). Good clinical care guidelines and guidelines as per the Helsinki Declaration were followed throughout the process.

**Data collection & participants:** We opted the total enumeration method for case recruitment. All patients admitted to our institute within the period of July to August 2022 were considered for the study. Hospitalized patients with radiologically confirmed acute ischemic stroke showing acute brain infarct(s) on MR or CT imaging of the brain were approached for the study. The study population consisted of 78 patients of acute ischemic stroke. 

Inclusion criteria: Adult patients (age > 18 years), radiologically (MR or CT brain imaging) confirmed & hospitalized cases of acute ischemic stroke, and examination, radiology and blood sampling completed within 24 hours of hospitalization. Exclusion Criteria: Pregnant women and minors (age < 18 years). The IDs of patients presenting to the medicine wards in July and August 2022 were fed into www.randomizer.orgfor randomization to reduce bias.

After taking written informed consent from the guardian and the patient (wherever possible), neurological examination was done to ascertain the NIHSS score of the patient according to the guide available on the National Institutes of Health website. Muir et al.’s study (4), as mentioned before, helped to gauge prognosis of patients based on baseline NIHSS scores. Accordingly, we adopted a general classification criterion wherein we graded the severity of stroke as minor/mild if the NIHSS score lay between 1-4, moderate between 5-14, moderate-to-severe between 15-20, and severe between 21-42. Fasting blood samples were collected and tested for serum HCY, fasting blood glucose (FBG) and lipid profile, which consisted of triglycerides (TG), total cholesterol (TCHOL), high-density lipoprotein cholesterol (HDLC) and low-density lipoprotein cholesterol (LDLC).

Ultrasonography of the neck was done to ascertain common carotid artery (CCA) IMT and internal carotid artery (ICA) IMT using the Color Doppler method. We also noted the percentage of luminal narrowing (% LN) caused by plaques, if any. Ultrasound were performed by several radiologists; however, a common protocol for measurement was followed, e.g. CCA IMT measured on the far (posterior) wall of mid-segment of CCA in longitudinal view to reduce operator-to-operator variation.

**Statistical analysis:** Data were recorded using Microsoft Office Excel 2016 spreadsheet software and was further analyzed on IBM SPSS Version 26 software. Normality tests, paired and independent samples t-tests, receiver-operator characteristic (ROC) curves etc. were used to analyze the data on SPSS.

## Results

**Figure 1:**
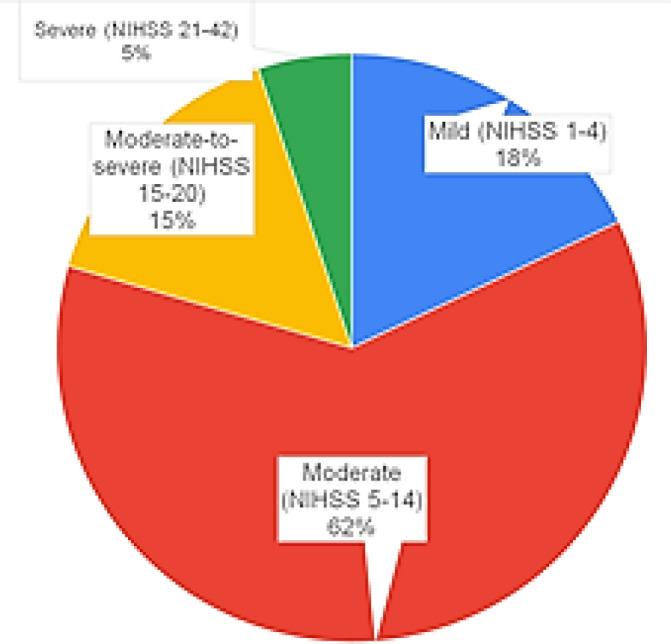
Proportion of cases graded by stroke severity according to NIHSS score

Out of the 78 stroke patients, 44 were males and 34 were females. χ2 (chi-square) test performed to assess the pattern of distribution, returned a non-significant *p*-value. Hence, the seemingly unequal distribution of males and females was not statistically significant. Average age of male and female subjects was 57.88 ± 13.97 and 59.16 ± 13.62 years respectively. Out of the 78 patients, 70 were diagnosed with acute ischemic infarcts on magnetic resonance (MR) modalities while 8 were diagnosed on computed tomography (CT) modalities. The average NIHSS score of all stroke patients was 10.07 ± 5.95 varying from 1 to 24. 14 (17.95%) patients presented with mild CVA, 48 (61.54%) patients with moderate CVA, 12 (15.38%) patients with moderate-to-severe CVA, and 4 (5.13%) patients with severe CVA (figure 1). 22 (28.07%) patients were euhomocysteinemic (EHcyc) while 56 (71.93%) patients were hyperhomocysteinemic (HHcyc). 

19 out of 78(24.36%; 15 males & 4 females) cases showed hyperlipidemic lipid profiles. Cases were classified as hyperlipidemic if at least one of these criteria was fulfilled: (i) TCHOL > 240 mg/dL, (ii) TG > 200 mg/dL, (iii) HDLC < 40 mg/dL, (iv) LDLC > 160 mg/dL**. **

**Grouping by severity of stroke using NIHSS Score**

Independent samples t-test was applied to all measured parameters, and patients were grouped separately as minor/mild stroke, moderate, moderate-to-severe and severe cases based on NIHSS scores as described in the introduction. Here onwards,‘t’ refers to the test statistic of independent samples t-test and ‘*p*’ refers to the significance expressed as the p-value. LDLC was significantly higher (t = 2.074; *P*= 0.043*) in cases with NIHSS score ≥ 5 compared to those with NIHSS score < 5.

**Figure 2:**
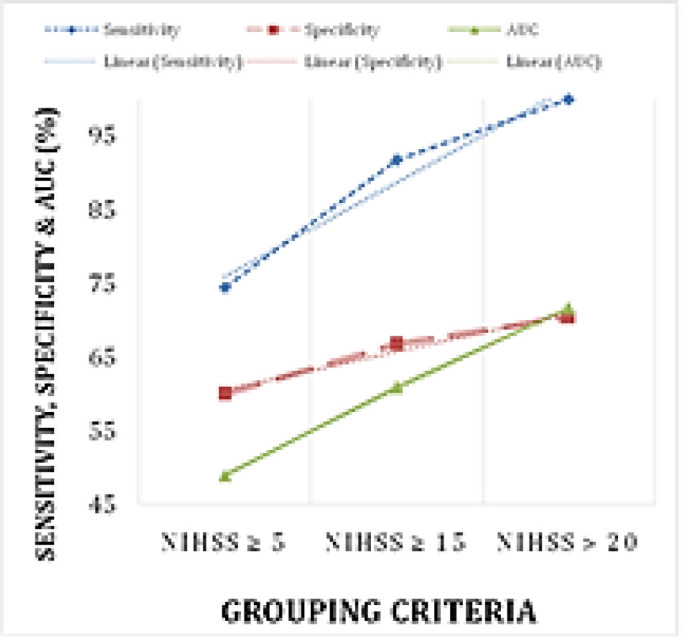
Predictive value of HCY for NIHSS score via ROC parameters (sensitivity, specificity and AUC)

Receiver-operator characteristic (ROC) analysis was performed three times by successively grouping cases as having positive state defined by NIHSS ≥ 5, NIHSS ≥ 15 and NIHSS > 20. Area under curve (AUC) of HCY increased linearly from 49.0% to 71.6%. Keeping the HCY cutoff at 15 μmol/L, the sensitivity increased linearly from 74.5% to 100.0% while the specificity increased linearly from 60.0% to 70.4% (figure. 2). HCY cutoff of ≥ 15μmol/L had 91.7% sensitivity & 66.7% specificity for predicting NIHSS ≥ 15. This result is interpreted as confirming that the HHcyc state holds a good predictive value for predicting the severity of stroke as classified by the NIHSS score, and is most valuable for NIHSS ≥ 15 (i.e. moderate-to-severe and severe cases).

**Grouping by serum HCY levels: EHcyc v/s HHcyc patients**

The data were grouped as either EHcyc patients with HCY < 15 μmol/L or HHcyc subjects with HCY ≥ 15 μmol/L.

**Figure 3:**
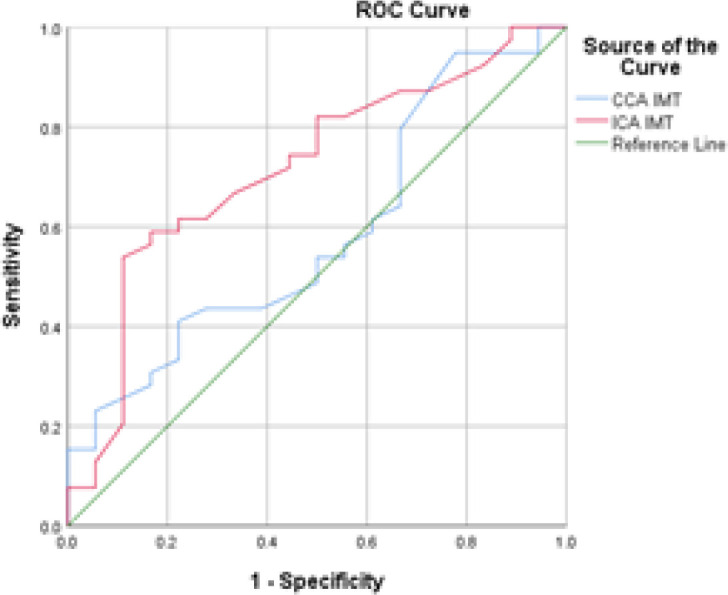
ROC analysis of HHcyc state with CCA IMT and ICA IMT

Independent samples t-tests were run to check differences between EHcyc and HHcyc patients. ICA IMT (t = 2.132; *P*= 0.039*) was significantly higher in HHcyc patients compared to EHcyc patients. Other parameters (even NIHSS score, CCA IMT etc.) were not significantly different between the two groups. ROC analysis was done to check the predictive value of measured parameters for HHcy. ICA IMT had the highest AUC (AUC > 0.7; *P*= 0.002*), having sensitivity and specificity significant at the 0.01 level. P-values of other parameters (including CCA IMT, conventional risk factors such as LDLC, and NIHSS score) were not significant regardless of their AUC. It is interesting to note here that ICA IMT apparently provides a more sensitive and specific prediction of the HHcyc state than CCA IMT, which is conventionally measured as *the* carotid IMT (figure 3).

**Grouping by ICA IMT > 1.0 mm and ≤ 1.0 mm.**

Independent samples t-test was applied to all measured parameters, and cases were grouped as having ICA IMT either > 1.0 mm or ≤ 1.0 mm. Cases with ICA IMT > 1.0 mm had higher HCY (t = 2.230; *P*= 0.030*), LDLC (t = 2.097; *P* = 0.041*), and CCA IMT (t = 4.200; *P*= 0.000*).

**Correlations of blood parameters**

The glycemic marker, FBG, was positively correlated with CCA IMT. Upon analyzing the lipid profile, TCHOL was found to be positively correlated with all other parameters of lipid profile (TG, HDLC, LDLC) as well as CCA IMT and ICA IMT. TG positively correlated with LDLC and NIHSS score (table 1). 

**Figure 4:**
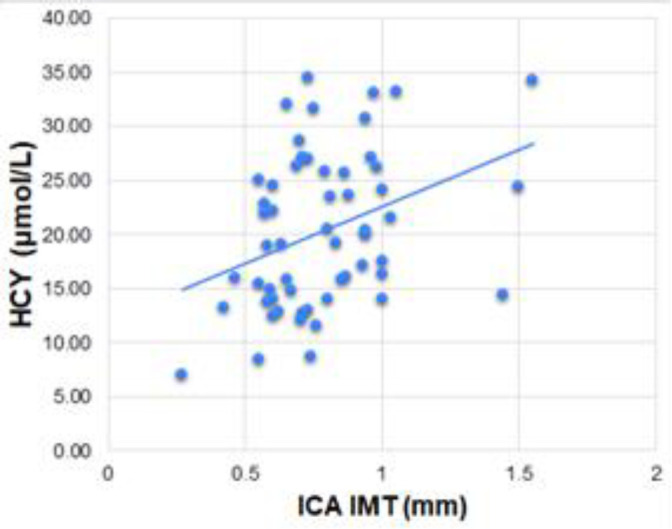
Scatter plot of ICA IMT against HCY

The main analyte, HCY, was found to be positively correlated with ICA IMT (figure 4; table 1). It was interesting to note that it had no other significant correlations, not even with CCA IMT or NIHSS score or any other blood parameters.

**Table 1:** Pearson correlations for pairs of analytes

**Analyte pair**	**Pearson Correlation Coefficient**	***P*** **-value**	**Analyte pair**	**Pearson Correlation Coefficient**	***P*** **-value**
TCHOL-CCA IMT	0.296	0.026*	HCY-ICA IMT	0.331	0.012*
TCHOL-ICA IMT	0.320	0.015*	TG-LDLC	0.350	0.008*
TCHOL-TG	0.337	0.010*	TG-NIHSS	0.262	0.049*
TCHOL-HDLC	0.666	0.000*	FBG-CCA IMT	0.286	0.031*
TCHOL-LDLC	0.947	0.000*			

TCHOL: Total Cholesterol, CCA IMT: Common Carotid Artery Intima-Media Thickness, ICA IMT: Internal Carotid Artery Intima-Media Thickness, TG: Triglyceride, HDLC: High Density Lipoprotein Cholesterol, LDLC: Low Density Lipoprotein Cholesterol, HCY: Homocysteine, NIHSS: National Institutes of Health Stroke Scale, FBG: Fasting Blood Glucose

## Discussion

Cerebral atherosclerosis is the basic underlying pathophysiology in ischemic stroke. Khan et al. (12) found that hyperlipidemia was present in 16% stroke patients, while the present study found hyperlipidemia in 24.36% of stroke patients. We report here that patients with NIHSS score ≥ 5 have higher LDLC compared to those with NIHSS score < 5. Affirming our result, Uno et al. (13) found that increased levels of oxidized LDLC correlated with larger extent of ischaemic lesions and could predict enlargement of the lesions. Our study reported that the glycemic marker, FBG, was positively correlated with CCA IMT. FBG has a well-documented incremental effect on the lipid profile. Hyperglycemia is essentially the cause of lipemic and cardiovascular morbidity in DM (14). Impaired lipemic control as well as pre-existing cardiovascular damage in DM contributes to increased carotid IMT. Brohall et al.’s systematic review (15) consisting of over 4,000 diabetic patients, and reporting higher IMT in diabetics than controls, gives excellent insight and evidence-based support to this finding.

Stein et al. (16) observed that hyperhomocysteinemia (HHcy) is associated with various vascular and hematological abnormalities such as endothelial injury, increased synthesis of thromboxane A2, activation of clotting factors V, X & XII, inhibition of antithrombin III & protein C, promotion of binding of lipoprotein(a) to fibrin, and growth of smooth muscle cells. All of these processes are known to be risk factors in the development and progression of atherosclerosis, leading to coronary artery disease, CVA, and peripheral arterial vascular disease. 

A prospective population-based cohort study with nearly 10 years of follow-up concluded that HCY is an independent risk factor for incident stroke in elderly persons (17). Several other studies have also postulated that elevated HCY is a strong and independent risk factor for vascular diseases including ischemic cerebral stroke (18). Yang et al. (19) aptly noted that other experiments exploring the mechanism of HCY-induced atherosclerosis had used HCY concentrations 100 times higher than human HHcyc concentrations. To overcome this, Yang et al. cultured endothelial cells in clinically relevant HHcyc concentrations of 20 - 50 µmol/L and hypothesized that HCY-induced hypomethylation leads to delayed recovery from endothelial injury. They found that HHcy dramatically inhibited thymidine incorporation (indicator of DNA synthesis) and proliferation in endothelial cells. HCY was identified as a unique, cell type specific, growth inhibitory factor at clinically relevant concentrations in endothelial cells. Endothelial cell injury is a hallmark of atherosclerosis. Therefore, growth inhibition of endothelial cells may represent an important mechanism for HCY-induced atherosclerosis.

While some older reviews in the general population (20) found no correlation between HCY levels and IMT, newer studies, specifically in stroke patients (21), have found that HCY levels and IMT are positively correlated. Further, we found that HHcyc state was associated with increased ICA IMT. In fact, our ROC analysis suggested that ICA IMT is the best predictor of the HHcyc state. Dietrich et al.'s study (22) ended up with similar results and noted that the ICA/bulb segment is more prone to plaque formation. Hence, correlations with HCY at the proximal ICA or carotid bulb might suggest or confirm the detrimental mechanisms of HHcy.

We also found that the HHcyc state holds a good predictive value for severity of stroke as graded by the NIHSS score. As it turns out, not only does HCY predict NIHSS at presentation, but it also predicts early neurological deterioration (23) and CVA recurrence (24). We must correlate two of our results at this point. On one hand, patients with ICA IMT > 1.0 mm had higher HCY. On the other hand, HHcyc state was associated with higher ICA IMT. These two seemingly overlapping results suggest that ICA IMT > 1.0 mm tends to be associated with HHcy. Perhaps, if carotid Doppler scans reveal ICA IMT > 1.0 mm in a stroke patient, then prescribing a homocysteine test may be unnecessary and physicians could assume HHcy in such a case. Traditional parameters such as cholesterol levels, vices i.e. smoking etc., and systemic disorders like DM, HTN etc. have generally been deemed sufficient to evaluate the risk, severity and prognosis of CVA. However, several newer studies, including the one conducted by Fisher et al. (25), have noted that homocysteine-lowering therapy with higher doses of B-complex vitamins had benefits such as reduction of lipoprotein(a) and fibrinogen (26), and halting the progression of carotid plaque in patients whose plaque was progressing despite treatment of traditional risk factors. Spence (27) also suggested that higher doses of vitamin B12 and novel approaches to lowering serum homocysteine besides routine vitamin therapy could reduce the risk of stroke. Pyridoxine, folate and cobalamin, all of which have dietary origins, are three main cofactors in HCY metabolism. Deficiencies of these vitamins are rampantly prevalent in developing countries and may account for many cases of hyperhomocysteinemia and increased risk of stroke (28). 

Sainani et al. (29) noted that homocysteine-lowering therapy i.e. vitamins B6, B9 and B12 in combination with atorvastatin halted the progression of carotid plaque completely while atorvastatin alone was able to merely slow, but not halt, plaque progression. Vitamins have an indispensable role to play in the treatment of stroke as the risk of recurrence can be minimized if plaque progression is arrested early on. We have discussed at length the deleterious effects of HHcy and the role it plays in the development and progression of carotid plaque. However, the rampant prevalence of subclinical vitamin B12 and folate deficiency, especially in Indians, is a major hurdle in overcoming HHcy. At present, assaying vitamins or HCY is expensive and clinically impractical, as far as the Indian scenario is concerned. Therefore, as ICA IMT seems to predict HHcy, carotid Doppler protocol should include measurement of the same. If it is found to be > 1.0 mm and/or NIHSS ≥ 15 then measuring HCY may be unnecessary. Prospective prevention studies have shown that increased IMT is a powerful predictor of complications of stroke. However, IMT measurement requires methodological standardization before routine measurement of IMT can be implemented in clinical practice as a diagnostic tool for assessing cardiovascular risk in primary prevention and for gauging aggressiveness of treatment decisions (30). One limitation of our study was the smaller sample size; to apply the results to the whole population, cohort studies with larger sample size are required. Our study concluded that HHcyc state holds a reasonably good predictive value for predicting the severity of stroke. Stroke management protocols in India currently already include imaging techniques such as CT/MRI of brain and its vasculature as well as cervical ultrasound (i.e. Carotid Color Doppler). 

As we found that ICA IMT measurement via Doppler scan predict higher HCY levels, it may reduce the need to run a homocysteine test; this will reduce the burden on resources and time. We suggest that estimating HCY and measuring ICA IMT should be made part of the standard protocol for management of CVA and treatment regimens should plan long term follow-ups using these parameters as indicators of improvement alongside traditional investigations.
